# Lacanian Concept of Desire in Analytic Clinic of Psychosis

**DOI:** 10.3389/fpsyg.2017.00563

**Published:** 2017-04-11

**Authors:** Julieta De Battista

**Affiliations:** ^1^Laboratorio de Investigaciones en Psicoanálisis y Psicopatología, Facultad de Psicología, Universidad Nacional de La PlataBuenos Aires, Argentina; ^2^Consejo Nacional de Investigaciones Científicas y TécnicasBuenos Aires, Argentina; ^3^Comisión de Investigaciones Científicas de la Provincia de Buenos AiresBuenos Aires, Argentina

**Keywords:** desire, psychosis, psychoanalysis, Lacan, knot, jouissance, body, Joyce

## Introduction

The conceptof desire is central to Lacan's theory and practice, even if it is not among the four fundamental concepts of psychoanalysis—unconscious, *Trieb*, repetition, transference, it can be understood that it underlies all of them. The concept of desire is inherent to the ethics of psychoanalysis that Lacan formulated, therefore it is especially concerned with a practice whose operation is defined by the function of analyst's desire. However, this central thesis of Lacan has been called into question with regard to psychosis. Some Lacanian scholars have derived from the foreclosure of the Name-of-the-Father a lack of desire in psychosis.

This paper aims to discuss the relative absence of references to the concept of psychotic desire in Lacanian schools. The debate is relevant because Lacan did exclude neither desire nor psychosis from his conception of analytic treatment.

It is frequent that in the transmission of the approach of this type of cases into the Lacanian schools, the concept of desire is not used, but rather the consequences of its absence are emphasized (De Battista, 2012). For example, in two of the latest publications compiled by Miller, where there are more than 20 clinical cases of psychosis treated by Lacanian analysts, the concept of desire is not evoked to think about the changes caused by the cure. In the clinical cases where this concept is mentioned, the authors highlight that desire has not operated (cf. Borie, 2011; Dewambrechies-La Sagna, 2011; Di Ciaccia, 2011; Zerghem, 2011; Klotz, 2012; Magnin et al., 2012).

In the argumentation of these authors, this non-operational desire would go hand in hand with intrusive and invasive phenomena that would account for a delocalized jouissance, whose restraint would depend on its fixation through identifications, delusional metaphors or writing practices, introducing a limitation of jouissance (Maleval, 2000; Soria Dafunchio, 2008; Miller, 2011, 2012).

Other authors claim that desire would not be absent in psychosis (Lombardi, 1992; Soler, 2004), nonetheless it is restricted to paranoia, declaring its abolition in schizophrenia (Quinet, 2006). However, even in those cases where psychotic desire is considered, the affirmation of its existence does not go hand in hand with a clarification of its operation in the cure. The authors again resort to the idea of an invasion of jouissance, which should be limited (Soler, 1987; Quinet, 2006; Soria Dafunchio, 2008; Miller, 2011, 2012; Redmond, 2013). The notion of limitation of jouissance is that which is most frequently used to account for the analytical treatment of psychosis (Maleval, 2000).

In view of the current state of affairs with regard to the subject treated, the following questions deserve—in my view—an investigation.

Firstly, Is desire the exclusive patrimony of those clinical types derived from the *père-version* (neurosis/perversion)? What would be the Lacanian arguments to sustain the absence of desire in psychosis? Secondly and according to my hypothesis of the importance of psychotic desire, which kind of desire would operate in psychosis?

## Lacanian concept of desire

On his return to Freud, the Lacanian perspective reintroduces the question of desire as the basis of analytic experience. Desire and unconscious go hand in hand for Freud. The Freudian notion of desire is early linked to the effort toward motility and the difference between what is found and what is sought: negativity and lack that drive the indestructible pursuit of desire.Unconscious desire is then the core of our being. Desire irrigates us, innervates us and includes that vital and sexual dimension. It seems to be the way by which *Trieb*, thanks to the institution of a fault, works in the unconscious, thus becoming a kind of *Trieb* destiny, a treatment of the real of the body.

Lacan (1966a) pointed out that the question of desire remained veiled in the conceptualizations of analytic experience. He proposed to reintroduce it in terms of an ethic that is not that of Aristotle—which exiles the desire to be beyond the domain of reason—but it is rather in harmony with the purposes of Spinoza, who conceives desire as the essence of human kind. A journey through references, brief and metonymic as our object seems to demand, reminds us that for Lacan desire is also linked to the vital impulse and to libido (Lacan, 1986, 1971–1972). Desire cannot be said, it is manifested in the interval, in the interstices and defined for Lacan as the metonymy of being in the subject, or the metonymy of the lack in being (Lacan, 2013). This definition is maintained throughout the entirety of his teaching. Even in 1975, Lacan argues that the unconscious determines the subject as being, being that disappears in the metonymy in which the desire is supported, impossible to say as such (Lacan, 1974–1975). The desire can be articulated but it is not articulable, it is irreducible to the demand and the necessity, cannot be named, cannot be sifted, it is of the order of the unconscious fault (Lacan, 1976–1977). However, desire can be clinically verified (Lacan, 2005).

For Lacan, desire is established in the dialectic of the fault. The Other gives the subject an experience of his desire which is the basis of the position in the structure. This implies a certain dependence on the desire of the subject with respect to the desire of the Other, whereby the desire for desire is the essential dimension (Lacan, 1986). The subject is born into language and is already determined in his unconscious by the desire of the Other, it is born of a desire (Lacan, 1971–1972, 1974–1975). The point is to have been desired, that is what we found in the analytic experience, even for those to whom that experience was perturbed in their constitution.

## Neurotic position and psychotic position with respect to desire

The relation of the subject's desire to the Other's desire is not a structure reserved solely for neurosis. Lacan (2013) is explicit in this respect, when he says that “it is an essential structure, not only of neurosis, but of every analytically defined structure” (p. 502). He does not renounce to situate the position of desire in different structures; there would then be different forms of desire and different subjective structures. The relations of desire become the field where analytic experience is articulated and this implies an ethic of desire characteristic of psychoanalysis, an ethics that sets the question of the analyst's desire. Analysts are intermediaries, they preside over the advent of desire, they are a kind of midwives of desire (Lacan, 2013).

If we return to the different subjective structures and the different forms of desire, we find that for the neurotic, whose position in desire is the fantasy, the metaphorical reference to the Name-of-the-Father knots the registers and establishes an oedipal psychic reality, therefore religious. The object *a*, cause of desire, is trapped in the center of the knot. The desire is mediated by the phallic reference that gives it a common measure and symbolizes the X of the mother's desire. The function of the father knots desire to a law, that of the interdiction of incest: here is the *père-version* (Lacan, 1974–1975). The X of desire is fixed on the fantasy that brings an interpretation. The neurotic subject has a relation to desire by the way of fantasy, due to the fact that fantasy has the function of sustaining desire.

Notably, the situation is different for the psychotic subject, because his condition implies the rejection of the father's metaphorical reference, that is, the foreclosure of the Name-of-the-Father, a circumstance of the subjective position for Lacan (1966b). But the absence of the father's metaphor does not condition the presence of desire, whose support is not metaphor but metonymy. Consequently, if the Name-of-the-Father has been rejected, the metaphorical effect in this point does not occur and the X of the maternal desire is not symbolized by the signifier of the phallus: which is why the desire of the Other is not symbolized (Lacan, 1998).

According to Lacan (1986), the desire of the mother is the founding desire of the whole structure, and in the psychotic subject it is outside of the symbolization introduced by the paternal metaphor, therefore the knotting of records does not occur in an Oedipal way. This argument is not enough to say that there is no desire in the psychotic, but rather that it is a not symbolized desire, without the reference that introduces the phallus as a signifier of the lack.

Evidently, Lacan's intention has not been to exile desire from the field of psychosis. What is in question is the reference that desire can find in the signifier of lack, the phallus, but not the existence of desire itself. Then, the question would not be that of the absence of desire in psychosis, but that of a desire which is not symbolized by the Name-of-the-Father. That is, a desire that is not knotted to the law of the father, dimension that characterizes the position of the psychotic as one of rejection of the paternal imposture. The desire of the psychotic would not be sealed by the consent of the father.

A close bond between desire and the law of the father has veiled that for Lacan desire is an absolute condition. That is to say, desire is not relative to another thing, it is detached from the dependence to something else. Desire makes the law and not the law which introduces the desire. Desire is autonomous with regard to the mediation of the law, the law itself originates from desire and not vice versa (Lacan, 1966c). A certain transmission effect has reversed this assertion, by concluding that the law of the Father is the one that introduces the desire, and then it follows that the psychotic's rejection of the Name-of-the Father implies an exclusion from desire. But Lacan did not make the Name-of-Father or the law the absolute condition, but desire.

In this way, the question of desire remains in Lacan beyond the father, it concerns the condition of the speaking being in language and does not necessarily entail the operation of a metaphorical reference. The desire offers a key to read what could knot real, symbolic, and imaginary without reference to the Name-of-the-Father and without this being constituted in a deficient condition, but simply different.

Desire is an absolute condition, not relative to something else, desire is the essence of the human being. This thesis is maintained throughout the teaching of Lacan: from the 50s to the late 80s, the desire is at the heart of the analytic experience as a possible *Triebschicksale* (Lacan, 1980). In 1962, Lacan affirms that the specificity of psychosis with respect to desire is that, in its structuring, the psychotic does not know the phallus and the Other, therefore the body acquires all the importance. One example of this thesis could be found in Cotard's syndrome where Lacan (1978) recognized a desire of death. However, this one is not the only form of psychotic desire that Lacan places, in 1974–1975 he also speaks of frozen desire in paranoia (Lacan, 1974–1975).

## Conclusion

However, the position on the desire differs in neuroses, psychosis, and perversion. In the case of psychotic desire, Lacan has affirmed a fundamental relationship with the body (Lacan, 1961–1962). The references worked allow us to propose that the problem is not a lack of desire in psychosis, but rather its support. We can determine delusional supports of desire, as in Schreber's case, or symptomatic supports of desire as in Joyce's solution “desire to be an artist who will keep the critics busy for three hundred years” (Lacan, 2005: 88). Both of them have a common term: the asymptotic character. One possible clinical application of these references is to question the position of the psychotic in desire. From this perspective, the analyst's position is relevant because of its specificity: become the cause of the analysand's desire, a sort of “tailored *partenaire*” for the psychotic, who finds it difficult to sustain his desire.

## Author contributions

The author confirms being the sole contributor of this work. She has designed the conception of the article, made the analysis and interpretation of sources, drafted the paper and revised and approval final version to be published.

## Funding

This work was supported by the Consejo Nacional de Investigaciones Científicas y Técnicas (CONICET), the Universidad Nacional de La Plata (UNLP) and the Comisión de Investigaciones Científicas (CIC).

### Conflict of interest statement

The author declares that the research was conducted in the absence of any commercial or financial relationships that could be construed as a potential conflict of interest.
